# Impaired learning and memory in CD38 null mutant mice

**DOI:** 10.1186/s13041-016-0195-5

**Published:** 2016-02-09

**Authors:** Somi Kim, TaeHyun Kim, Hye-Ryeon Lee, Eun-Hye Jang, Hyun-Hee Ryu, Minkyung Kang, So-Young Rah, Juyoun Yoo, Bolam Lee, Jae-Ick Kim, Chae Seok Lim, Sang Jeong Kim, Uh-Hyun Kim, Yong-Seok Lee, Bong-Kiun Kaang

**Affiliations:** Department of Biological Sciences, College of Natural Sciences, Seoul National University, 1 Gwanangno, Gwanak-gu, Seoul 08826 South Korea; Department of Life Science, Chung-Ang University, Seoul, 156-756 South Korea; Departments of Biochemistry, Institute of Cardiovascular Research, Chonbuk National University Medical School, Jeonju, 561-182 South Korea; Department of Physiology, Seoul National University College of Medicine, Seoul, 110-799 South Korea

## Abstract

CD38 is an enzyme that catalyzes the formation of cyclic ADP ribose and nicotinic acid adenine dinucleotide phosphate, both of which are involved in the mobilization of Ca^2+^ from intracellular stores. Recently, CD38 has been shown to regulate oxytocin release from hypothalamic neurons. Importantly, CD38 mutations are associated with autism spectrum disorders (ASD) and CD38 knockout (CD38^−/−^) mice display ASD-like behavioral phenotypes including deficient parental behavior and poor social recognition memory. Although ASD and learning deficits commonly co-occur, the role of CD38 in learning and memory has not been investigated. We report that CD38^−/−^ mice show deficits in various learning and memory tasks such as the Morris water maze, contextual fear conditioning, and the object recognition test. However, either long-term potentiation or long-term depression is not impaired in the hippocampus of CD38^−/−^ mice. Our results provide convincing evidence that CD38^−/−^ mice show deficits in various learning and memory tasks including spatial and non-spatial memory tasks. Our data demonstrate that CD38 is critical for regulating hippocampus-dependent learning and memory without modulating synaptic plasticity.

**Electronic supplementary material:**

The online version of this article (doi:10.1186/s13041-016-0195-5) contains supplementary material, which is available to authorized users.

## Background

CD38 is an ADP-ribosyl cyclase that catalyzes the formation of cyclic ADP ribose (cADPR) and nicotinic acid adenine dinucleotide phosphate, which are involved in the mobilization of Ca^2+^ from intracellular stores [1–3]. Intracellular calcium release plays a critical role in regulating neuronal functions including neurotransmitter release and synaptic plasticity [4]. Recently, CD38 has been shown to regulate oxytocin (OXT) release from hypothalamic neurons by modulating intracellular Ca^2+^ mobilization [5, 6]. OXT and Ca^2+^ secretion in response to depolarization is decreased in CD38 null mutant mice [6].

Studies have shown that CD38^−/−^ mice display autism spectrum disorder (ASD)-like behavioral phenotypes that can be reversed with OXT treatment [6, 7] and mutations in CD38 are associated with ASD in human patients [8, 9]. It is well known that learning disability is common in those with an ASD [10]. Although it is known that OXT neurons in the hypothalamus send their projections to the hippocampus wherein OXT receptors are highly expressed [11–13] and that OXT regulates hippocampal synaptic plasticity [14, 15], it remains largely unknown whether CD38 deletion affects hippocampal synaptic plasticity and hippocampus-dependent learning and memory.

Here, we report that CD38^−/−^ mice show deficits in various learning and memory tasks such as the Morris water maze, contextual fear conditioning, object recognition, and social recognition tests. These findings indicate that CD38 is critically involved in regulating hippocampus-dependent learning and memory.

## Results

### Hippocampus-dependent memory is impaired in CD38^−/−^

**Fig. 1:**
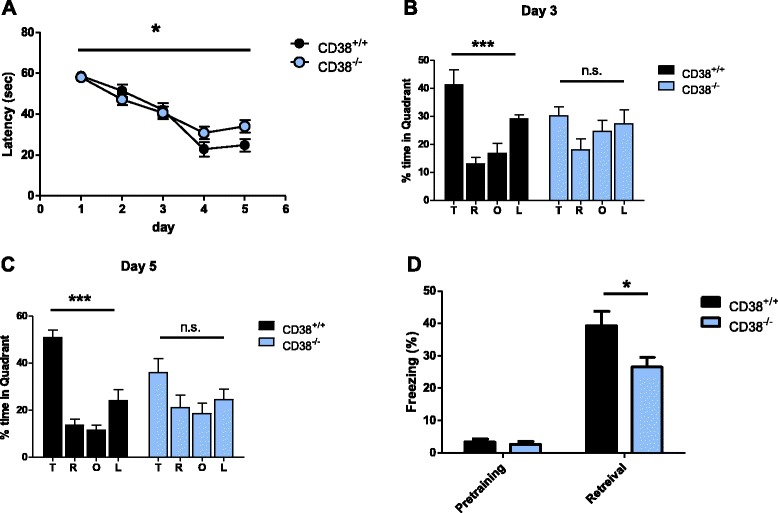
CD38^−/−^ mice show impaired hippocampus-dependent learning and memory. **a** The learning curve during 5 training days of the Morris water maze task showing the latency for the mice to reach the target platform. (CD38^+/+^, *n* = 9; CD38^−/−^, *n* = 13) **b** Time spent in each quadrant during a 1-min probe test after training sessions on training day 3. T: target, R: right, O: opposite, L: left quadrant. **c** Time spent in each quadrant during a 1-min probe test after training sessions on training day 5. T: target, R: right, O: opposite, L: left quadrant. **d** Freezing levels of CD38^+/+^ (black) and CD38^−/−^ (blue) mice before (pre-training) and 24 h after (retrieval) contextual fear conditioning (CD38^+/+^, *n* = 9; CD38^−/−^, *n* = 13). n.s., not significant. All graphs are plotted using means ± SEM

To examine whether deletion of CD38 affects learning and memory, we tested CD38^−/−^ mice in the Morris water maze test, which requires intact hippocampal function [16, 17]. In this task, mice are trained to learn and remember the location of the hidden platform beneath the surface of the water by using spatial cues. The knockouts took significantly longer to reach the hidden platform compared to their wild-type (WT) littermates in the training trials [CD38^+/+^, *n* = 9; CD38^−/−^, *n* = 13; two-way analysis of variance (ANOVA), interaction between genotype and day, F_4, 344_ = 2.529, * *p* < 0.05; Fig. 1a]. Importantly, the average swimming speed was not different between genotypes (CD38^+/+^, 16.14 ± 1.06 cm/s, *n* = 9; CD38^−/−^, 16.99 ± 0.83 cm, *n* = 13, unpaired *t*-test, *p* = 0.5281), suggesting that the knockouts show comparable motor activity to WT mice. Furthermore, CD38^−/−^ mice showed comparable locomotive activity and anxiety level to WT controls in the open field and elevated plus maze tasks (Additional file 1: Figure S1). Spatial memory for locating the platform was examined in the probe trials during which the platform was removed from the pool and the mice were allowed to search for the platform. In two probe trials performed at the end of the 3rd and 5th day training trials, WT mice showed specific memory for the target quadrant in which the platform was located during the training (day 3, one-way ANOVA; F_3, 24_ = 9.921, ***p < 0.001; day 5, one-way ANOVA, F_3, 24_ = 22.82, ***p < 0.0001; Fig. 1b and c). However, CD38^−/−^ mice did not show any significant preference for the target quadrant (day 3, one-way ANOVA, F_3, 36_ = 1.228, *p* = 0.3137; day 5, one-way ANOVA, F_3, 36_ = 1.742, *p* = 0.1758; Fig. 1b and c), suggesting that CD38 deletion impairs spatial learning and memory in mice.

Next, we examined the knockout mice in the contextual fear conditioning task, which is another hippocampus-dependent memory task [18]. In the training session, mice were placed in a conditioning chamber where mild foot shocks were delivered. Twenty-four hours after the training, the mice were exposed to the same conditioning chamber and their freezing behavior was assessed. Consistent with the results from the Morris water maze task, CD38^−/−^ mice showed significantly less freezing than WT (CD38^+/+^, n = 10; CD38^−/−^, n = 9; unpaired *t*-test, *p < 0.05; Fig. 1d), demonstrating that hippocampal learning and memory are impaired in CD38^−/−^ mice.

### Social and nonsocial recognition memory are impaired in CD38^−/−^

**Fig. 2:**
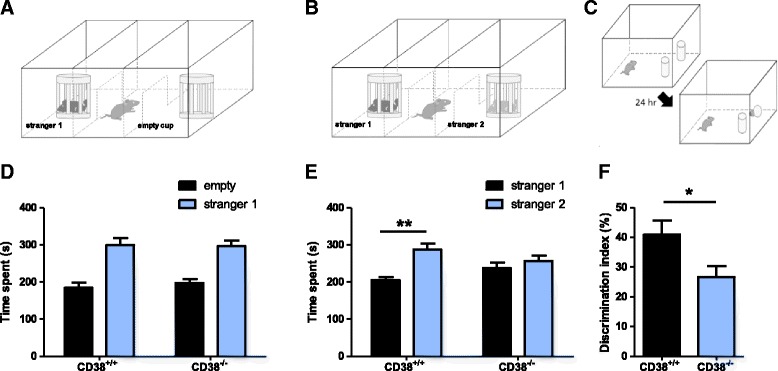
Social and nonsocial recognition memory is impaired in CD38^−/−^ mouse. **a-b** Experimental design for 3-chamber tests measuring sociability (**a**) and social recognition memory (**b**). **c** Experimental design for novel object recognition memory test. **d** Exploration time of CD38^+/+^ and CD38^−/−^ mice for the empty cup (black) and the cup with the stranger mouse (blue). (CD38^+/+^, *n* = 9; CD38^−/−^, *n* = 9). **e** Exploration time of CD38^+/+^ and CD38^−/−^ mice for the cup with the familiar mouse (black) and the cup with the stranger mouse (blue). **f** Discrimination index of CD38^+/+^ (black) and CD38^−/−^ (blue) mice (CD38^+/+^, *n* = 7; CD38^−/−^, *n* = 10). All graphs are plotted using means ± SEM

It has been shown that parental behavior and social memory are impaired in CD38^−/−^ mice [6, 7]. Additionally, OXT gene or OXT receptor deletion show impaired social recognition in mice [19–21]. We used the three-chamber test to examine the sociability and social memory of CD38^−/−^ mice [22]. Animals with normal sociability show a preference for the cup with a stranger mouse. The results indicated that CD38^−/−^ mice showed comparable social interaction to WT littermates (CD38^+/+^, *n* = 9; CD38^−/−^, *n* = 9; two-way ANOVA; interaction between genotype and cup (empty/social), F_1, 16_ = 0.1463, *p* = 0.7072; Fig. 2a, d). During the social memory task, WT mice spent significantly more time exploring the novel stranger mouse. However, CD38^−/−^ mice failed to distinguish the novel stranger mouse from the familiar mouse (CD38^+/+^, *n* = 9, paired *t*-test, stranger vs. familiar, **p < 0.01; CD38^−/−^, *n* = 9; paired *t*-test, stranger vs. familiar, *p* = 0.5223; Fig. 2b, e).

Next, we examined whether CD38 deletion affects nonsocial recognition memory by performing the novel object recognition test, which is widely used to examine non-spatial recognition memory in rodents [23, 24]. This task exploits the preference for a novel object over a familiar one, which is a typical trait in mice. The level of preference to the novel object is measured as an indicator of memory. CD38^−/−^ mice exhibited lower levels of preference for the novel object at the retention phase compared with WT controls (CD38^+/+^, *n* = 7; CD38^−/−^, *n* = 10; unpaired *t*-test for discrimination index; *p < 0.05; Fig. 2c, f). These results show that CD38 deletion affects both social and nonsocial recognition memory in mice.

### CD38^−/−^ mice show normal synaptic plasticity

**Fig. 3:**
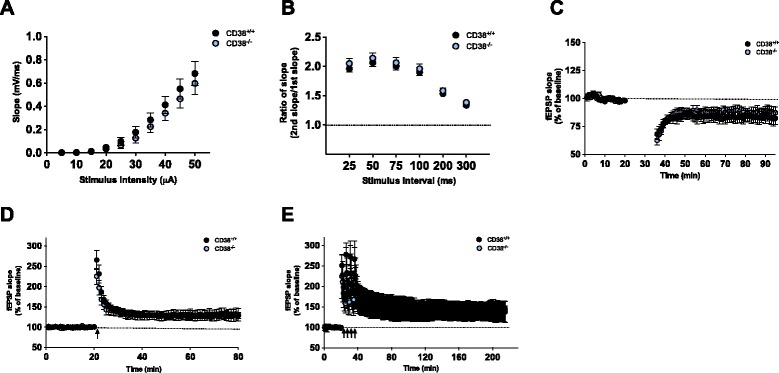
Normal basal synaptic transmission and synaptic plasticity in CD38^−/−^ mice. **a** Input–output relationships at Schaffer collateral-CA1 synapses are comparable between CD38^−/−^and wild-type littermates (CD38^+/+^, *n* = 18; CD38^−/−^, *n* = 12). **b** Paired pulse ratio is normal in CD38^−/−^ mice (CD38^+/+^, *n* = 14; CD38^−/−^, *n* = 10). **c** NMDAR-dependent LTD at Schaffer collateral-CA1 synapses in CD38^+/+^ and CD38^−/−^ mice are comparable. **d** High frequency stimulation (HFS)-induced E-LTP at Schaffer collateral -CA1 synapses in CD38^+/+^ and CD38^−/−^ mice are comparable. **e** L-LTP is not impaired at Schaffer collateral-CA1 synapses in CD38^−/−^ mice

To identify the mechanism responsible for the memory deficit caused by CD38 deletion, we examined electrophysiological properties of CD38^−/−^ mice by performing extracellular field recordings at the Schaffer collateral (SC)–CA1 synapse in acute hippocampal slices. Input–output relationship and paired-pulse facilitation (PPF) ratios were indistinguishable between WT and CD38^−/−^ mice (Input–output, CD38^+/+^, *n* = 18; CD38^−/−^, *n* = 12; Repeated measure two-way ANOVA, effect of genotype, F_1,252_ = 0.4931, *p* = 0.4883, Fig. 3a; PPF, CD38^+/+^, *n* = 14; CD38^−/−^, *n* = 10; Repeated measure two-way ANOVA, effect of genotype, F_1,110_ = 0.5807, *p* = 0.4541, Fig. 3b), demonstrating that the genetic deletion of CD38 does not affect basal synaptic transmission.

It has been previously shown that cADPR is critically involved in hippocampal LTD by stimulating Ca^2+^ release from ryanodine-sensitive stores in the presynaptic neuron [25]. Since CD38 is an enzyme that catalyzes cADPR synthesis, we first examined NMDAR-dependent LTD at the SC–CA1 synapse by delivering low frequency stimulation (900 pulses at 1 Hz) in acute hippocampal slices. There was no significant difference in the level of LTD between genotypes (average of fEPSP slopes for last 5 min; CD38^+/+^, *n* = 6, 83.2 ± 5.9%; CD38^−/−^, *n* = 8, 88.0 ± 5.1%; unpaired *t*-test; *p* = 0.5520; Fig. 3c), indicating that the genetic deletion of CD38 does not affect NMDAR-LTD in the hippocampus.

LTP at SC–CA1 synapse plays an important role in spatial learning and memory [26, 27]. Mutant mice with impaired LTP often show deficits in hippocampus-dependent learning and memory [26, 27]. However, CD38^−/−^ mice showed comparable level of early-phase LTP (E-LTP) induced by a single pulse of high frequency (100 Hz) stimulation to WT controls (average of fEPSP slopes for last 5 min; CD38^+/+^, *n* = 6, 129.4 ± 7.3 %; CD38^−/−^, *n* = 6, 130.1 ± 14.7 %; unpaired *t*-test; *p* = 0.9689; Fig. 3d). Consistently, theta-burst stimulation (TBS)-induced E-LTP was not different between genotypes (average of fEPSP slopes for last 5 min; CD38^+/+^, *n* = 7, 157.4 ± 4.4 %; CD38^−/−^, *n* = 6, 171.5 ± 6.1 %; unpaired *t*-test; *p* = 0.0808; Additional file 1: Figure S2). Late-phase LTP (L-LTP) is a form of synaptic plasticity that is dependent on *de novo* protein synthesis and is considered a mechanism for long-lasting memory [28]. We induced L-LTP in the hippocampal slices by delivering four pulses of high frequency tetanus in 5 min intervals and found that CD38^−/−^ mice showed similar level of L-LTP compared to WT littermates (average for last 10 min; CD38^+/+^, *n* = 10, 143.6 ± 7.1 %; CD38^−/−^, *n* = 9, 138.6 ± 8.3 %; unpaired *t*-test; *p* = 0.6531; Fig. 3e). This result shows that the genetic deletion of CD38 does not affect LTP in hippocampal SC–CA1.

## Discussion

CD38^−/−^ mice showed deficits in parental and social behaviors and furthermore, CD38 mutations have been associated with human autism spectrum disorder (ASD) [6–9, 29]. The first report of the identification of single nucleotide polymorphisms (SNPs) in the CD38 gene in patients with ASD suggested that CD38 mutations might be associated with high functioning ASD [9]. However, it is well known that many individuals with ASD have co-occurring cognitive problems including intellectual disability [10]. Moreover, a recent study showed that a genetic deletion involving CD38 is associated with mild learning disability [8], suggesting that CD38 deficiency may result in intellectual deficits in addition to the ASD-related phenotypes. In the present study, we demonstrate that the genetic deletion of CD38 affects not only social behavior, but also learning and memory in mice in object recognition, contextual fear conditioning and Morris water maze task.

A previous study suggested that spatial learning in the Morris water maze is not impaired in CD38^−/−^ mice based on results that used the time taken to find the hidden platform during the training phase, without performing probe trials [30]. However, rigorous criterion for learning during the Morris water maze task is dependent upon performance during the probe trial [31] and we found that CD38^−/−^ mice show deficits during the training and probe trials. In addition, we found that CD38^−/−^ mice show deficits in other learning and memory tasks including contextual fear conditioning, object recognition, and social recognition tests, demonstrating that both spatial and non-spatial memory are impaired in CD38^−/−^ mice. Interestingly, it was reported that CD38^−/−^ mice showed normal learning in the passive avoidance test, suggesting that only specific forms of learning are regulated by CD38-mediated signaling [6].

Synaptic plasticity is considered a cellular mechanism for learning and memory, and we examined whether synaptic plasticity is impaired in the hippocampus of CD38^−/−^ mice [26]. However, neither LTP nor LTD is altered in the hippocampal SC-CA1 area of CD38^−/−^ mice. These results raise three possibilities regarding the cellular and molecular mechanisms responsible for the learning and memory deficits in CD38^−/−^ mice. First, plasticity in brain areas other than the hippocampus may be affected in CD38^−/−^ mice. For instance, perirhinal cortex is critically involved in object recognition memory that is impaired in CD38^−/−^ mice [32, 33]. Second, it is possible that a dysregulation in intracellular Ca^2+^ homeostasis and signaling in the hippocampal or cortical cells in CD38^−/−^ mice is responsible for the memory deficits. CD38 is an ADP-ribosyl cyclase that regulates the intracellular Ca^2+^ concentration and mobilization [1–3]. Calcium is a second messenger critically involved in regulating synaptic plasticity and subsequently regulating learning and memory [34]. Although we could not detect any significant changes either in basal synaptic transmission or in short-term plasticity in the hippocampus of CD38^−/−^ mice, we cannot exclude the possibility that other cellular processes that are sensitive to intracellular Ca^2+^ might be altered in CD38^−/−^ mice. Finally, CD38 deletion may disrupt information processing without affecting synaptic plasticity in the brain. OXT secretion from hypothalamic neurons and axon terminals has been shown to be reduced in CD38^−/−^ mice [6]. A recent study showed that OXT enhances signal-to-noise ratio in the hippocampus by modulating the activity of fast-spiking interneurons [11]. Thus, it is plausible to postulate that genetic deletion of CD38 reduces OXT level and may affect information processing in the hippocampus or other brain areas by disrupting the fidelity of spike transmission and spike timing, which subsequently impairs learning and memory [6, 11]. It would be of interest to examine whether CD38 deletion has any impact on the activities of fast-spiking interneurons and furthermore to test whether increasing OXT level could rescue the memory deficits in CD38^−/−^ mice. Identifying the precise mechanism underlying learning and memory deficits in CD38^−/−^ mice would help developing therapeutic target for the learning deficits that occur in individuals with ASD.

## Conclusions

Our results provide converging evidence that CD38^−/−^ mice are impaired in various learning and memory tasks including spatial and non-spatial memory tasks. This is the first report demonstrating that CD38 is a critical regulator for hippocampus-dependent learning and memory.

## Materials and Methods

### Mice

Male CD38^−/−^ and wild-type (WT) littermates with a C57BL/6J genetic background were used for the behavioral experiments. The Animal Care and Use Committee of Seoul National University approved the animal protocols.

### Behavior tests

For all behavior tasks, male mice aged 8–15 weeks old were used and before performing the task, mice were put on a shelf for at least 40 min for stabilization.

#### Morris water maze

Mice were handled for 3 min at the same time for 7 consecutive days before performing the test. When handling was over, mice were put into a gray opaque cylinder shaped tank (140 cm diameter, 100 cm height) placed in a room with multiple spatial cues including a water tap, and a computer desk where the experimenter sits. The tank was divided into 4 virtual quadrants and a 10 cm diameter-platform was placed at the center of a quadrant (TQ). Other 3 quadrants were named by their position from TQ. The tank was filled with water (20 ~ 22 °C) until the water level was 1 cm higher than the platform and white paint was added. Before the first training trial of each mouse on training day 1, mice were placed on the platform for 30 s. On training days, mice were released at the edge of the maze facing the inner wall of the tank and trained to reach the platform for 60 s. Releasing point was randomly chosen at each trial. When the mice failed to reach the platform, they were guided to or placed on the platform for 10 s and were rescued from the maze. When the mice successfully reached the platform and stayed on the platform more than 1 s, mice were rescued from the maze after 10 s. Mice were trained with 4 trials per one training day and the trial interval between trial 1 and 2 or trial 3 and 4 was 1 min and between trial 2 and 3 was 30 ~ 45 min. Every mouse received 4 training trials per day for 5 consecutive days. Probe tests were performed in the same condition with training trials except the absence of the platform and the mice were tracked for 1 min with a tracking program (EthoVision 3.1; Nodulus). Probe test 1 was performed on training day 3 after all training trials were completed. Probe test 2 was performed 24 h after the training trials of training day 5.

#### Contextual fear conditioning test

Prior to contextual fear conditioning, 3-min handling per each mouse was performed for 4 consecutive days. When handling was over, mice were trained for contextual fear conditioning. Mice were placed in the chamber for 3 min. After 148 s they were presented with an unsigned foot shock (2 s, 0.8 mA). Then mice were returned to the chamber for fear memory test after 24 h. Freezing (immobile posture except respiration) level was measured automatically by a computer program (Freeze Frame; Coulbourn).

#### Three-chamber test

Stranger mice were handled for 3 min and then habituated in a wired cup placed in the 3-chamber apparatus for 5–10 min for 4 consecutive days. When the handling was over, the test mice were habituated to the 3-chamber apparatus for 10 min with the doors opened. When the habituation was completed, the test mouse was guided to the center chamber and the doors were closed. A wired cup with stranger 1 mouse and empty cup (for sociability test) were introduced into the other two chambers, and then opened the doors. The movement of the test mouse was tracked for 10 min with a tracking program (EthoVision 3.1; Nodulus). When the sociability test session was over, the test mouse was guided to the center chamber and the doors were closed. Another stranger mouse (stranger 2) was introduced into the empty cup (for social recognition test). The doors were opened and the movement of the subject mouse was tracked for another 10 min with the same tracking program. For each set of experiment, the orientation of two wired cups containing stranger 1 or stranger 2 (or empty) was counterbalanced.

#### Novel object recognition (NOR) test

Mice were handled for 3 min for 4 consecutive days before performing NOR test. The task was performed in an open field box, which was made of opaque acryl (40 × 40 × 40 cm). Object A was a light bulb and object B was a glass vial and there were no differences in preference between objects [AT(Time spent exploring object A)/ BT(Time spent exploring object B) = 0.9678 ± 0.0773, *n* = 4, one sample *t*-test, theoretical mean = 1, *p* = 0.7049]. Each object height was 10 cm, hard for mice to climb to object during task. On the day of sample phase, mice were placed to the open field box with two identical objects for 15 min. On retention phase test, which was performed 24 h after sample phase, mice were placed to the arena again for 10 min with two different objects (one was the same object presented at the sample phase, the other was a novel object newly presented at the retention phase). The time spent for the mice exploring each object was recorded. The discrimination index was calculated as the difference (AT-BT) in time spent by each mouse exploring the novel object compared with the familiar object divided by the total time spent exploring both of two objects (AT + BT). Preference was derived from the exploration time to a novel object divided by the total time spent exploring both of two objects.

### Electrophysiology

Transverse hippocampal slices (400 μm thick) for field excitatory postsynaptic potential (fEPSP) recordings were prepared as previously described [35]. The SC pathway was stimulated every 30 s using concentric bipolar electrodes (MCE-100; Kopf Instruments). Field potentials were amplified, low-pass filtered (GeneClamp 500; Axon Instruments), and then digitized (NI PCI-6221; National Instruments) for measurement. Data were monitored, analyzed online and reanalyzed offline using WinLTP program. For LTP and LTD experiments, after a stable baseline was recorded, high frequency stimulation (100 Hz, 1 s for HFS-LTP), four trains of high frequency stimulation (4 x 100 Hz, 1 s each, 5 min intertrain interval for HFS-L-LTP), low frequency stimulation (1 Hz, 900 stimuli for LFS-LTD), or theta burst stimulation (3 x TBS, 1 s each for TBS-LTP) was delivered.

### Statistics

For water maze data, we used one-way ANOVAs to analyze quadrant occupancy (% time spent in quadrant). When two groups were compared, unpaired or paired two-tailed *t*-test was used. LTP data were analyzed by using unpaired two-tailed *t*-test on the average of the last 5 min (E-LTP) or 10 min (L-LTP) of recordings. All the data are represented as mean ± SEM.

## Additional file

Additional file 1: Figure S1. CD38^−/−^ mouse shows normal locomotion and basal anxiety. (A) CD38^+/+^ (black) and CD38^−/−^ (blue) mice spent comparable time at the center of the open field (CD38^+/+^, *n* = 7; CD38^−/−^, *n* = 10; unpaired *t*-test, *P* = 0.272). (B) CD38^+/+^ (black) and CD38^−/−^ (blue) mice showed similar level of anxiety assessed in the elevated plus maze test (CD38^+/+^, *n* = 12; CD38^−/−^, *n* = 9; two-way ANOVA, interaction F (1, 2) = 0.119, *p* = 0.888). (C) Deletion of CD38 gene did not cause any decrease in total distance moved in the open field test (CD38^+/+^, *n* = 7; CD38^−/−^, *n* = 10; unpaired *t*-test, *p* = 0.366). All graphs were plotted mean ± SEM. **Figure S2.** CD38^−/−^ mouse shows normal TBS-induced E-LTP. TBS-induced E-LTP at SC-CA1 synapses showed no difference in WT and CD38^−/−^mice (CD38^+/+^, *n* = 7, 157.4 ± 4.4 %; CD38^−/−^, *n* = 6, 171.5 ± 6.1 %; unpaired *t*-test, *p* = 0.0808, n.s.). (PDF 124 kb)

## Abbreviations

ADP: Adenosine diphosphate
ACSF: Artificial cerebrospinal fluid
ASD: Autism spectrum disorders
CA1: Cornu ammonis 1
cADPR: Cyclic ADP ribose (cADPR)
E-LTP: Early-phase LTP
fEPSP: Field evoked postsynaptic potential
HFS: High-frequency stimulation
L-LTP: Late-phase LTP
LTD: Long-term depression
LTP: Long-term potentiation (LTP)
LFS: Low-frequency stimulation
NMDAR: N-methyl-D-aspartate receptor
NOR: Novel object recognition (NOR)
OXT: Oxytocin (OXT)
PPF: Paired-pulse facilitation
SC: Schaffer collateral
SNPs: Single nucleotide polymorphisms
TBS: Theta-burst stimulation
WT: Wild-type
