# Supplementary figures and images for: Impaired learning and memory in CD38 null mutant mice
Source: Mol Brain. 2016 Feb 9;9:16. doi: 10.1186/s13041-016-0195-5 (PMC4746819; doi:10.1186/s13041-016-0195-5)

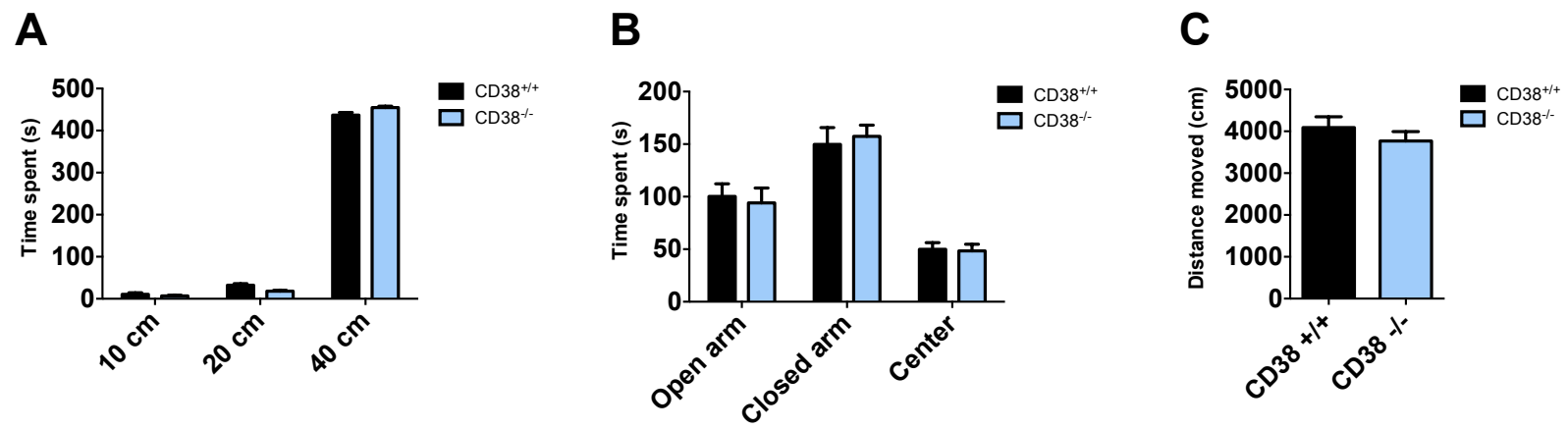

Supplementary Figure 1

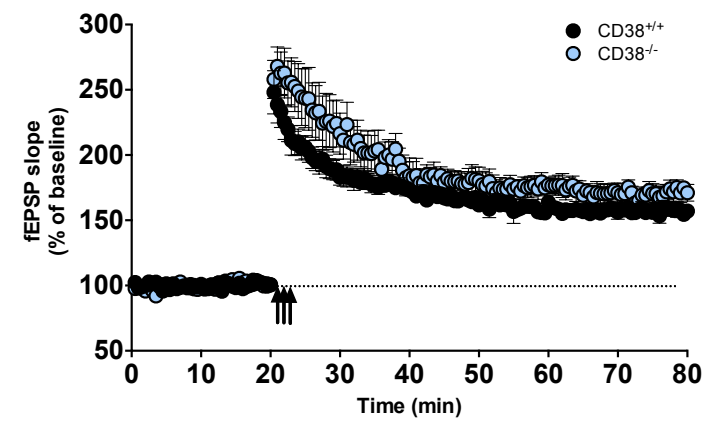

Supplementary figure 2

Supplement: Additional file 1: Figure S1. — CD38−/− mouse shows normal locomotion and basal anxiety. (A) CD38+/+ (black) and CD38−/− (blue) mice spent comparable time at the center of the open field (CD38+/+, n = 7; CD38−/−, n = 10; unpaired t-test, P = 0.272). (B) CD38+/+ (black) and CD38−/− (blue) mice showed similar level of anxiety assessed in the elevated plus maze test (CD38+/+, n = 12; CD38−/−, n = 9; two-way ANOVA, interaction F (1, 2) = 0.119, p = 0.888). (C) Deletion of CD38 gene did not cause any decrease in total distance moved in the open field test (CD38+/+, n = 7; CD38−/−, n = 10; unpaired t-test, p = 0.366). All graphs were plotted mean ± SEM. Figure S2. CD38−/− mouse shows normal TBS-induced E-LTP. TBS-induced E-LTP at SC-CA1 synapses showed no difference in WT and CD38−/−mice (CD38+/+, n = 7, 157.4 ± 4.4 %; CD38−/−, n = 6, 171.5 ± 6.1 %; unpaired t-test, p = 0.0808, n.s.). (PDF 124 kb) [file 13041_2016_195_MOESM1_ESM.pdf]
